# Effect of Antibiotic Incorporation on the Porosity of Polymethylmethacrylate (PMMA) Bone Cement: A Comparative Analysis

**DOI:** 10.7759/cureus.117846

**Published:** 2026-10-13

**Authors:** Paraskevas Georgoulas, Efthymios Iliopoulos, Ioannis Vasios, Athanasios Ververidis, Konstantinos Tilkeridis, Evanthia Perikleous, Nick Vordos, Georgios Drosos

**Affiliations:** 1 University Orthopaedic Department, University General Hospital of Alexandroupolis, Democritus University of Thrace, Alexandroupolis, GRC; 2 Academic Orthopaedic Department, University General Hospital of Alexandroupolis, Democritus University of Thrace, Alexandroupolis, GRC; 3 Orthopeadic Department, University General Hospital of Alexandroupolis, Democritus University of Thrace, Alexandroupolis, GRC; 4 Orthopaedic Surgery Department, University General Hospital of Alexandroupolis, Democritus University of Thrace, Alexandroupolis, GRC; 5 Paediatrics and Child Health Department, Nicosia New General Hospital, Nicosia, CYP; 6 Physics Department, School of Science, Biophysics Laboratory and Hephaestus Laboratory, Democritus University of Thrace, Kavala, GRC

**Keywords:** antibiotic-loaded pmma, bone cement, micro-computed tomography, pmma, pore architecture, raman spectroscopy, x-ray diffraction

## Abstract

Polymethylmethacrylate (PMMA) is widely applied in orthopedics. Antibiotic-loaded PMMA has been extensively used, and its mechanical impact, especially porosity, is of widespread importance. We assessed the effects of commercially available and add-on antibiotic PMMA in terms of porosity.

The study evaluated seven high-viscosity bone cement groups (S1-S7), with six specimens per group: an antibiotic-free control, three commercially antibiotic-loaded, and three manually antibiotic-loaded with gentamicin, vancomycin, clindamycin, or their combinations. Total porosity percentage was defined as the primary outcome, while open- and closed-pore parameters were secondary outcomes. Porosity was quantitatively evaluated using micro-computed tomography (μ-CT). Raman spectroscopy was used for complementary chemical characterization, and X-ray diffraction (XRD) was used for exploratory comparison of the relative crystalline contribution of the formulations.

μ-CT analysis showed that S1, S3, and S7 had the highest mean total and open porosity values, whereas S2, S4, and S6 demonstrated lower mean values. However, no significant between-group differences were detected for open-porosity or total-porosity percentages. Significant differences were identified for closed-pore number, volume, surface area, and percentage. S7 had the highest number of closed pores, while S4 showed a higher closed-pore volume than S2, S3, S5, and S6 (p < 0.05), a higher closed-pore surface area together with S7 than S1, S2, S3, S5, and S6 (p < 0.05), and a higher closed-pore percentage than S3 and S5 (p < 0.01). Raman spectra showed formulation-dependent spectral differences. Exploratory XRD indicated the highest relative crystalline contribution in S1 and the lowest in S7, without a consistent relationship with μ-CT porosity.

Conclusively, the tested formulations differed mainly in closed-pore architecture rather than in total or open porosity percentages. Raman spectroscopy provided complementary chemical information, whereas XRD showed descriptive differences in the crystalline contribution of the formulations. The mechanical and clinical relevance of microstructural findings requires further evaluation with mechanical and antibiotic-elution testing.

## Introduction

Polymethylmethacrylate (PMMA) is an acrylic plastic. It is formed through polymerization, an exothermic chemical reaction in which mixing a liquid monomer and a powder polymer leads to a hard cement [1,2]. Bone cement, primarily composed of PMMA, plays a crucial role in orthopedic surgery due to its mechanical strength and compatibility with antibiotic addition. Eventually, the trends of bone cement usage have evolved. Its current uses in general orthopedics consist of the management of infections, such as osteomyelitis, tumors, fractures, notably those with high risk for infection, such as complex open fractures, and, of course, arthroplasties.

Innovations, including antibiotic-loaded cements, have further extended its utility. Various antibiotics may be added to PMMA, although not all antibiotics are appropriate to be successfully mixed and used in bone cements. The fundamental necessity is that the mixable antibiotic can be heat-stable, elute over a proper time duration, and certainly must not significantly impact mechanical integrity and porosity [2-4].

Recent research has focused on optimizing antibiotic release and modifying the porosity of the cement matrix; increasing porosity enhances antibiotic elution as interconnected voids provide pathways for drug diffusion [5-7]. Regardless of the chosen loading technique, porosity can be altered by the application of a vacuum mixing system and, in vitro, by pore-forming agents, for instance, gelatin [8,9]. Noteworthy, porosity of the cured PMMA interferes with antibiotic elution as pores enlarge the cement's surface area [10]; however, increased porosity may compromise mechanical integrity, which is critical in load-bearing applications.

Micro-computed tomography (μ-CT) provides three-dimensional information on total porosity, pore distribution, pore volume, and pore connectivity [11]. Raman spectroscopy provides complementary information on the molecular structure and chemical composition of the PMMA matrix and may reveal formulation-dependent spectral changes [12-17]. Raman spectroscopy was not used in this study to quantify porosity or crystallinity. X-ray diffraction (XRD) was additionally performed to compare the relative contribution of broad amorphous scattering and sharp crystalline reflections in the complete cement formulations. Since PMMA is predominantly amorphous and sharp reflections may arise from crystalline radiopacifier or antibiotic phases, the XRD index was interpreted as a relative structural descriptor of the complete composite and not as absolute PMMA crystallinity [18,19]. As complementary objectives, Raman spectroscopy was used to characterize formulation-dependent chemical features, while exploratory XRD analysis assessed the relative crystalline contribution of the complete cement formulations. These techniques provided compositional and structural context for the μ-CT findings and were not used to quantify porosity.

The aim of this study was to compare total, open, and closed porosity, together with associated pore metrics, among seven PMMA bone cement formulations comprising an antibiotic-free control, manually antibiotic-loaded cements, and commercially antibiotic-loaded cements. The null hypothesis was that the measured porosity parameters would not differ among the tested formulations. Comparisons between manually and commercially loaded cements were interpreted in the context of differences in cement composition.

## Materials and methods

Seven groups of samples were used, each of which included six replicates, totaling 42 samples. Of these, in one group we did not add antibiotics, while in the remaining six groups gentamicin, vancomycin, clindamycin, or combinations thereof were added at different concentrations, as presented in detail in Table 1.

**Table 1:** Composition of bone cement samples

Samples	Description	Amount	Commercial Availability (Yes/No)	Added Antibiotic 1	Added Antibiotic 2
S1	PALACOS® R - High-Viscosity Bone Cement Without Antibiotics 1 x 40	-	Yes	-	-
S2	PALACOS® R - High-Viscosity Bone Cement Without Antibiotics 1 x 40	-	No	Gentamicin 0.5 g	
S3	PALACOS® R+G - High-Viscosity Bone Cement With Gentamicin 1 x 40	Gentamicin 0.5 g	Yes	-	-
S4	PALACOS® R - High-Viscosity Bone Cement Without Antibiotics 1 x 40	-	No	Gentamicin 0.5 g	Vancomycin 2 g
S5	COPAL® G+V - High-Viscosity Bone Cement With Gentamicin and Vancomycin 1 x 40	Gentamicin 0.5 g and Vancomycin 2 g	Yes	-	-
S6	PALACOS® R - High-Viscosity Bone Cement Without Antibiotics 1 x 40	-	No	Gentamicin 1 g	Clindamycin 1 g
S7	COPAL® G+C - High-Viscosity Bone Cement With Gentamicin and Clindamycin 1 x 40	Gentamicin 1 g and Clindamycin 1 g	Yes	-	-

All specimens were prepared strictly following the specified manufacturers’ instructions and then, as-mixed, immediately inserted into a specially designed and built moulding device in order to produce all necessary samples. Primarily, in order to prepare all the samples, we applied the PALAMIX® system (Heraeus Medical GmbH, certified under EU Medical Device Regulation standards, Wehrheim, Germany), a vacuum mixing system. Afterwards, we combined the powdered polymer and the liquid-stage monomer into the designated sterile cartridge, leading to a homogeneous material. Noteworthy, in antibiotic-loaded specimens, we also added antibiotic powder prior to accurate weighing using a high-precision scale (Ohaus Adventurer AR3130; OHAUS Corporation, Parsippany, NJ, USA). All specimens were prepared in an orthopedic operating room maintained at a constant ambient temperature of 22°C to simulate clinical preparation conditions. During the mixing phase, lasting 30 secs, we used consistent up-and-down strokes with a mixing rod, avoiding the introduction of air bubbles. When reaching a doughy, homogeneous cement consistency, we inserted it into a molding device with 75 mm x 10 mm x 3.3 mm dimensions [20]. An hour later were removed and transferred to room temperature, approximately 24°C, for a day.

Porosity analyses were performed using the Bruker μ-CT SkyScan 1278 (Bruker, Billerica, MA, USA), available at the Hephaestus Laboratory of the School of Sciences (Democritus University of Thrace). Scans were acquired with a 0.2° rotation step, an exposure time of 20 ms per step, and a total rotation of 360°. The X-ray source was set to 53 kV and 926 μA, and a 1 mm aluminum filter was applied to reduce beam hardening artifacts. Flat-field correction was carried out according to the manufacturer's instructions before the sections were performed. The step-and-shoot technique was selected. A total of 1.139 slides, each with a pixel size of 51.634 μm, were taken. Tomographic reconstruction was carried out using NRecon software (version 2.0.0.5; Bruker, Kontich, Belgium), while porosity analysis was conducted with CTAn (version 1.5.0; Bruker, Kontich, Belgium). Segmentation was performed using the automatic Otsu method, with a minimum threshold value of 70. Following binarization, the “remove black speckles” operation was applied to remove black components smaller than six voxels. The region of interest (ROI) was defined to encompass the entire specimen. Additionally, CTVox software (Bruker, Kontich, Belgium) was used to visualize structural and morphological features.

Statistical analysis of the μ-CT results was performed using IBM SPSS Statistics for Windows, Version 30 (Released 2024; IBM Corp., Armonk, New York, United States) [21]. The analysis of variance (ANOVA) method was conducted to examine whether there were differences in the mean values between the seven different sample groups. The Shapiro-Wilk and Levene tests were used to verify the normality of the results and the homogeneity of the variances, while statistical significance was set at p < 0.05. One-way ANOVA and Tukey post hoc testing were used on data that met the necessary conditions; otherwise, we applied the Kruskal-Wallis nonparametric test, followed by Dunn-Bonferroni or Steel-Dwass multiple comparisons for pairs of groups. For non-parametric data, effect size (ε^2^) was calculated, and the level of statistical significance was set at p < 0.05.

Raman spectra were acquired from the surface of each sample using a confocal Raman microscope (XploRA PLUS; Horiba Scientific, UK) equipped with a 785 nm diode laser source operated in edge filter mode. A 100× objective lens (VIS; Olympus Corporation, Tokyo, Japan) focused the excitation beam onto the sample surface. The Raman scattered light was passed through a 200 μm slit and collected via a spectrograph equipped with a 600 gr/mm grating centered at 750 nm and a 500 μm confocal pinhole. Spectral resolution was approximately 1-2 cm^-1^ across the scanned range of 200-1800 cm^-1^. Spectral acquisition was performed using LabSpec 6 software (Horiba Scientific, Edison, NJ, USA). Each spectrum was recorded as the average of five accumulations, with an integration time of two seconds per accumulation. All spectra were acquired in triplicate to ensure reproducibility.

XRD patterns were obtained using an X-ray diffractometer (D8 Focus; Bruker AXS GmbH, Karlsruhe, Germany) with copper Kα (Cu Kα) radiation (λ = 1.5406 Å), operated at 40 kV and 40 mA. Patterns were collected in locked-coupled geometry over the common range of 5°-70° 2θ, with a 0.1° step and a counting time of two seconds per step. The same processing procedure was applied across all patterns. A relative XRD crystalline contribution index was calculated as the area of the sharp diffraction features divided by the sum of sharp and broad corrected areas. This index was used only for descriptive comparison of the complete cement formulations and was not interpreted as an absolute PMMA crystallinity fraction. Only one XRD pattern was acquired per group (n = 1); therefore, the calculated index was used solely for exploratory, descriptive comparisons, and no inferential statistical analysis was performed.

## Results

We provide a brief overview of important parameters of pore structure, each revealing information about the internal architecture of the material, in order to facilitate the interpretation of the results. Firstly, the total porosity is presented, which encompasses all pores within the cement matrix, regardless of whether these pores are connected to the external surface or are isolated within the material [5]. This parameter is critical as it directly affects the mechanical properties, density, and behavior of the final cement. Secondly, we calculated the number of open pores, which refers to the number of pores that are directly or indirectly connected to the external surface of the cement sample [22]. Open pores form continuous pathways through the material and are critical for fluid transport, drug elution, and potentially nutrient diffusion in biological environments. Thirdly, the number of closed pores was calculated, which describes the total number of isolated voids that are completely enclosed within the matrix of the material and have no connection to the external surface [23]. Closed pores do not participate in fluid transport and can provide some resistance to external environmental changes. The ratio between open and closed pores significantly affects the mechanical integrity and long-term performance of the cement. Another parameter evaluated was the volume of open pores, which quantifies the total space occupied by all connected pores that form communication pathways with the external environment. This parameter directly affects the ability of the cement to deliver drugs and the rate of fluid penetration into the material. The volume of closed pores measures the total empty space occupied by the individual pores within the cement matrix. These closed voids can serve as stress concentration points and affect the overall density and mechanical response of the material under load. The open pore surface area represents the total interfacial area between the open pore network and the solid cement matrix. This parameter is particularly important in determining the contact surface available for biological interactions, protein adsorption, and cell adhesion. Finally, the closed pore surface area describes the internal surface of individual pores within the material. Although these pores do not come into direct contact with the external environment, their internal surfaces can affect the acoustic properties of the material and may provide points for internal stress concentration during mechanical loading [24].

Figure 1 shows representative μ-CT images of our samples. Indicatively, S1 shows intermediate density with several closed pores, S2 shows moderate porosity, and S3 shows a more homogeneous structure. S4 shows a microstructure with pore sizes larger than 3 mm, S6 shows high pore absorption, and S7 contains pores of about 1-2 mm in size.

**Figure 1:**
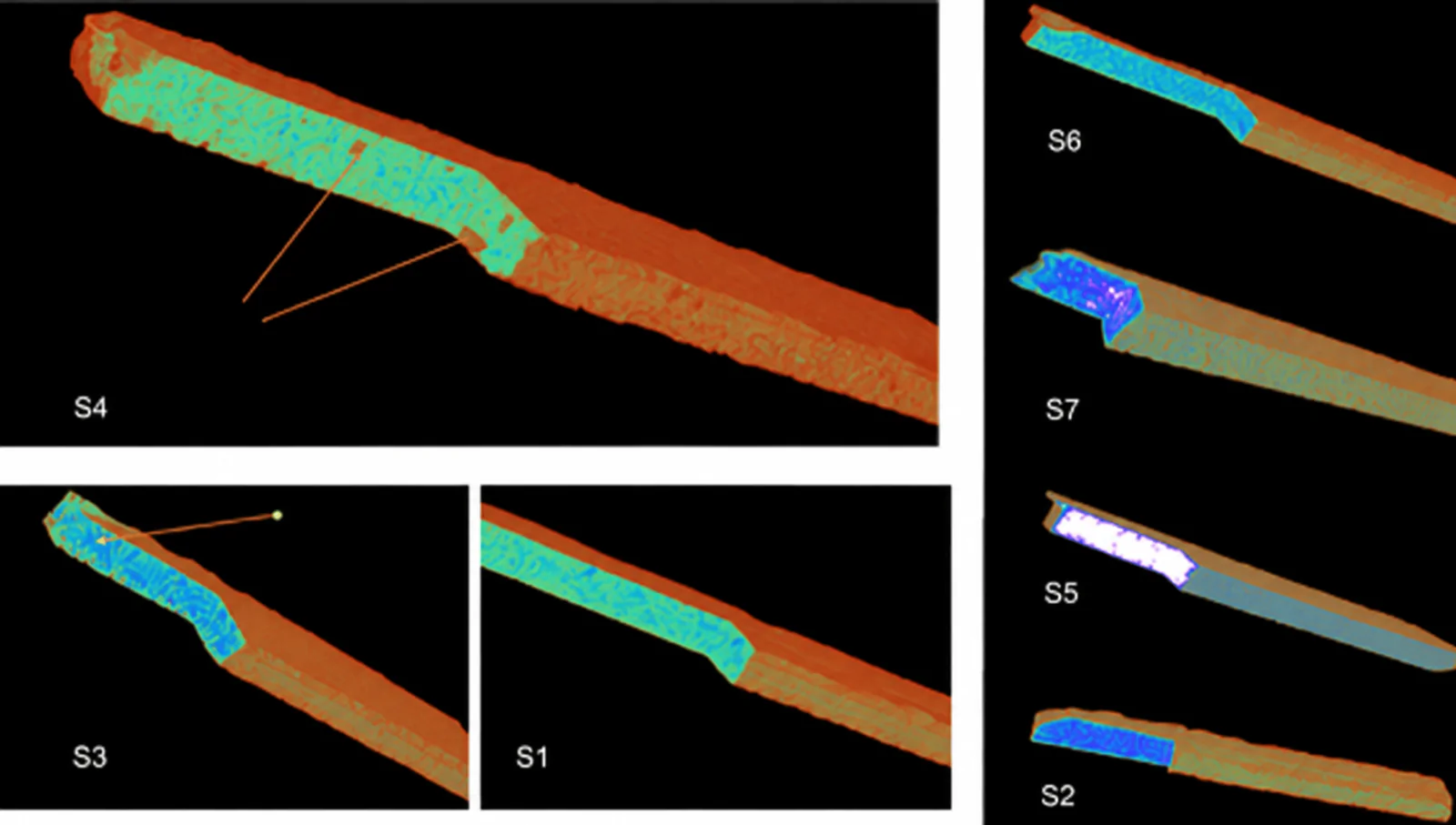
Representative 3D reconstructions of six bone cement groups available in the supplied source composite after renumbering The red arrows indicate the presence of pores.

μ-CT analysis provided data on porosity-related parameters, including the number, volume, and surface area of open and closed pores, as well as total porosity. Table 2 summarizes the mean ± SD values. Groups S1, S3, and S7 showed the highest total and open porosity, while S5 and S6 showed the lowest. Remarkably, S7, the commercial gentamicin-plus-clindamycin formulation, showed the largest mean number of closed pores.

**Table 2:** Summary of pore-related parameters (mean ± SD) per cement group

Group	Antibiotic	Number of Closed Pores	Volume of Closed Pores (mm^3^)	Surface of Closed Pores (mm^2^)	Closed Porosity (%)	Volume of Open Pores (mm^3^)	Open Porosity (%)	Total Pore Volume (mm^3^)	Total Porosity (%)
S1	Without Antibiotic Commercially	121.33 ± 71.20	7.40 ± 7.92	35.99 ± 23.03	0.27 ± 0.28	1831.02 ± 210.92	41.03 ± 4.91	1838.42 ± 214.74	41.04 ± 4.91
S2	Gentamicin 0.5 g Loaded	167.83 ± 59.62	5.26 ± 4.45	45.23 ± 29.33	0.15 ± 0.12	1608.26 ± 596.83	34.44 ± 8.88	1617.28 ± 602.21	34.58 ± 8.88
S3	Gentamicin 0.5 g Commercially	179.17 ± 149.92	2.75 ± 4.03	43.66 ± 66.17	0.12 ± 0.14	1337.72 ± 426.94	41.11 ± 12.46	1340.47 ± 431.02	41.98 ± 12.26
S4	Gentamicin 0.5 g +Vancomycin 2 g Loaded	602.83 ± 658.71	17.94 ± 10.48	209.20 ± 142.92	0.68 ± 0.34	1360.91 ± 418.73	34.14 ± 11.92	1378.85 ± 425.78	35.13 ± 13.05
S5	Gentamicin 0.5 g + Vancomycin 2 g Commercially	344.17 ± 131.86	1.89 ± 0.70	47.52 ± 15.94	0.07 ± 0.02	1648.13 ± 365.92	38.34 ± 3.93	1652.02 ± 365.92	38.98 ± 3.89
S6	Gentamicin 1 g + clindamycin 1 g Loaded	482.50 ± 479.61	5.12 ± 4.85	58.51 ± 32.85	0.19 ± 0.19	1407.36 ± 121.94	34.63 ± 2.79	1413.14 ± 121.94	34.81 ± 2.70
S7	Gentamicin 1 g + clindamycin 1 g Commercially	1802.33 ± 1100.57	5.66 ± 3.24	175.52 ± 112.46	0.21 ± 0.11	1774.20 ± 280.75	40.12 ± 2.50	1780.53 ± 281.81	40.48 ± 2.59

Figure 2 displays the Raman spectra of the seven representative formulations. All groups exhibited PMMA-related spectral features, with formulation-dependent differences in relative intensity and profile. Because absolute Raman intensity is affected by focusing, surface roughness, sampling location, and local heterogeneity, these differences were interpreted qualitatively and were not used to quantify porosity or crystallinity. The exact assignment of antibiotic-related bands was not considered conclusive because of the low antibiotic concentration and overlap with the PMMA spectrum [13-17].

**Figure 2:**
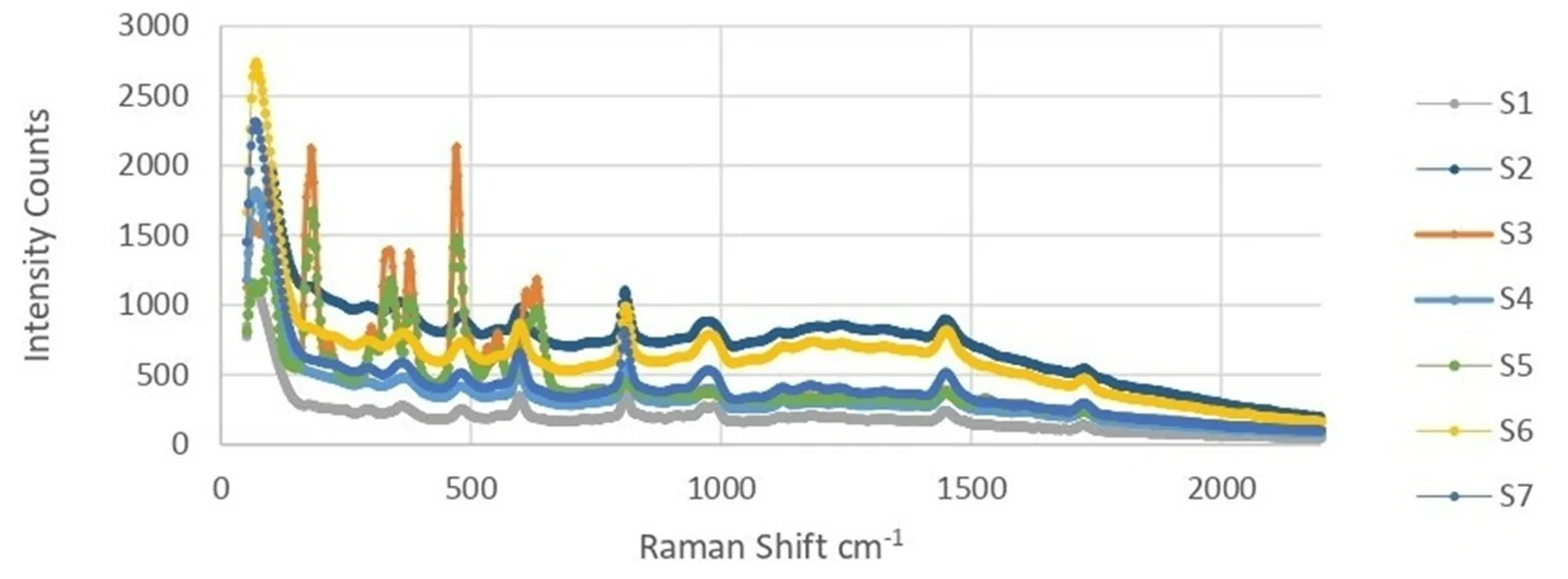
Raman spectra of bone cement samples

As shown in Figure 3, the relative XRD crystalline contribution index was 26.75% for S1, 21.11% for S2, 21.75% for S3, 21.67% for S4, 20.08% for S5, 21.43% for S6, and 13.92% for S7. S1 showed the highest and S7 the lowest descriptive value, while S2, S3, S4, and S6 were closely grouped. Because only one pattern was available per formulation and the sharp reflections may arise from crystalline radiopacifier and antibiotic phases, these results were treated as exploratory and no statistical significance was assigned.

**Figure 3:**
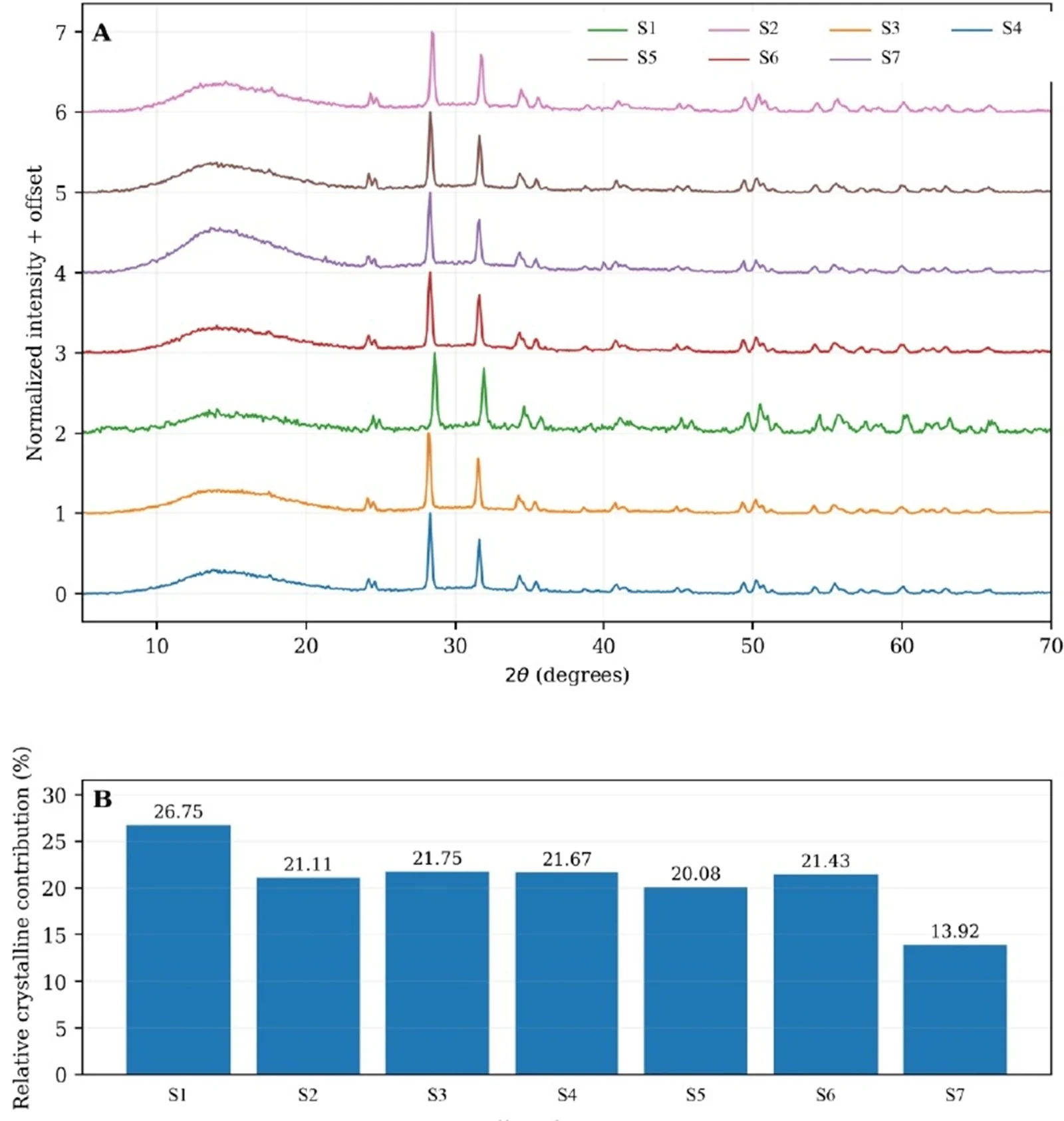
X-ray diffraction (XRD) analysis of the seven cement formulations (A) Background-corrected patterns normalized to their maximum intensity and vertically offset for visual comparison over the common range of 5°-70° 2θ. (B) The XRD crystallinity index was calculated from diffraction patterns relative to the total corrected scattering area. The index describes the crystallinity in the bulk material and does not represent the absolute crystallinity of PMMA. PMMA: polymethylmethacrylate

Significant between-group differences were identified for the number of closed pores (N_Closed) (H(6) = 22.561, p < 0.001), volume of closed pores (Vol_Closed) (H(6) = 15.135, p = 0.019), surface area of closed pores (Surf_Closed) (H(6) = 20.390, p = 0.002), and percentage of closed pores (Closed_Pct) (H(6) = 15.747, p = 0.015). Significant overall between-group differences were also observed for the volume of open pores (Vol_Open) (H(6) = 14.461, p = 0.025) and total pore volume (Total_Vol) (H(6) = 13.506, p = 0.036). In contrast, no significant differences were observed for open-porosity percentage (Open_Pct) (H(6) = 7.818, p = 0.252) or total-porosity percentage (Total_Pct) (H(6) = 7.714, p = 0.260). S7 had the highest N_Closed compared with all other groups (adjusted p < 0.05; Figure 4). S4 had a higher Vol_Closed than S2, S3, S5, and S6 (adjusted p < 0.05; Figure 5). Surf_Closed was higher in S4 and S7 than in S1, S2, S3, S5, and S6 (adjusted p < 0.05). S4 also showed a higher Closed_Pct than S3 and S5 (p < 0.01; Figure 6).

**Figure 4:**
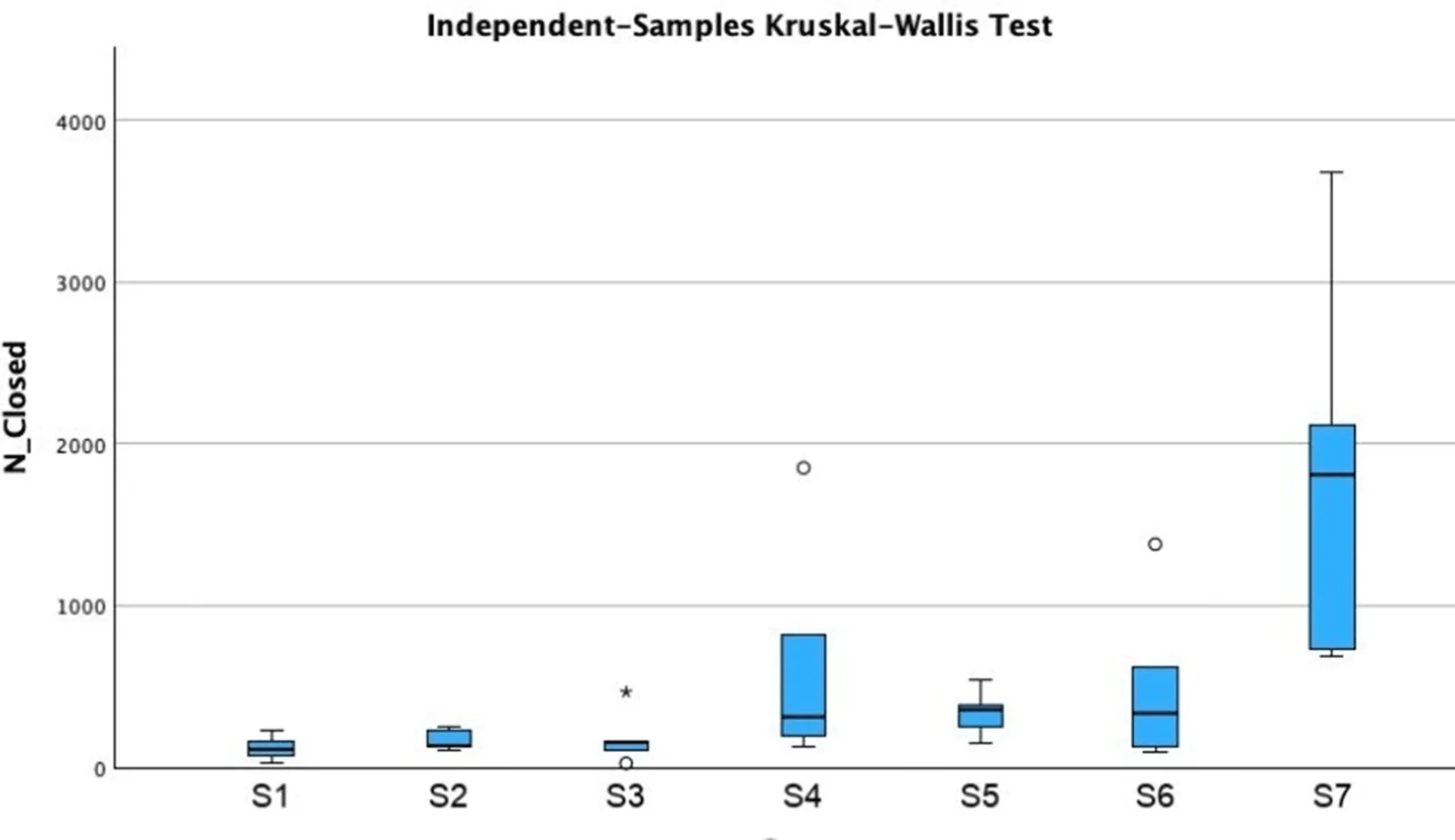
Box-and-whisker plot of closed-pore count (N_Closed) for groups S1-S7 in the revised order The Kruskal-Wallis test indicated between-group differences (H(6) = 22.561, p < 0.001), with S7 showing the highest values. Boxes show the middle 50% of values, and the horizontal line marks the median. Circles and asterisks indicate outliers and extreme outliers, respectively, rather than statistical significance.

**Figure 5:**
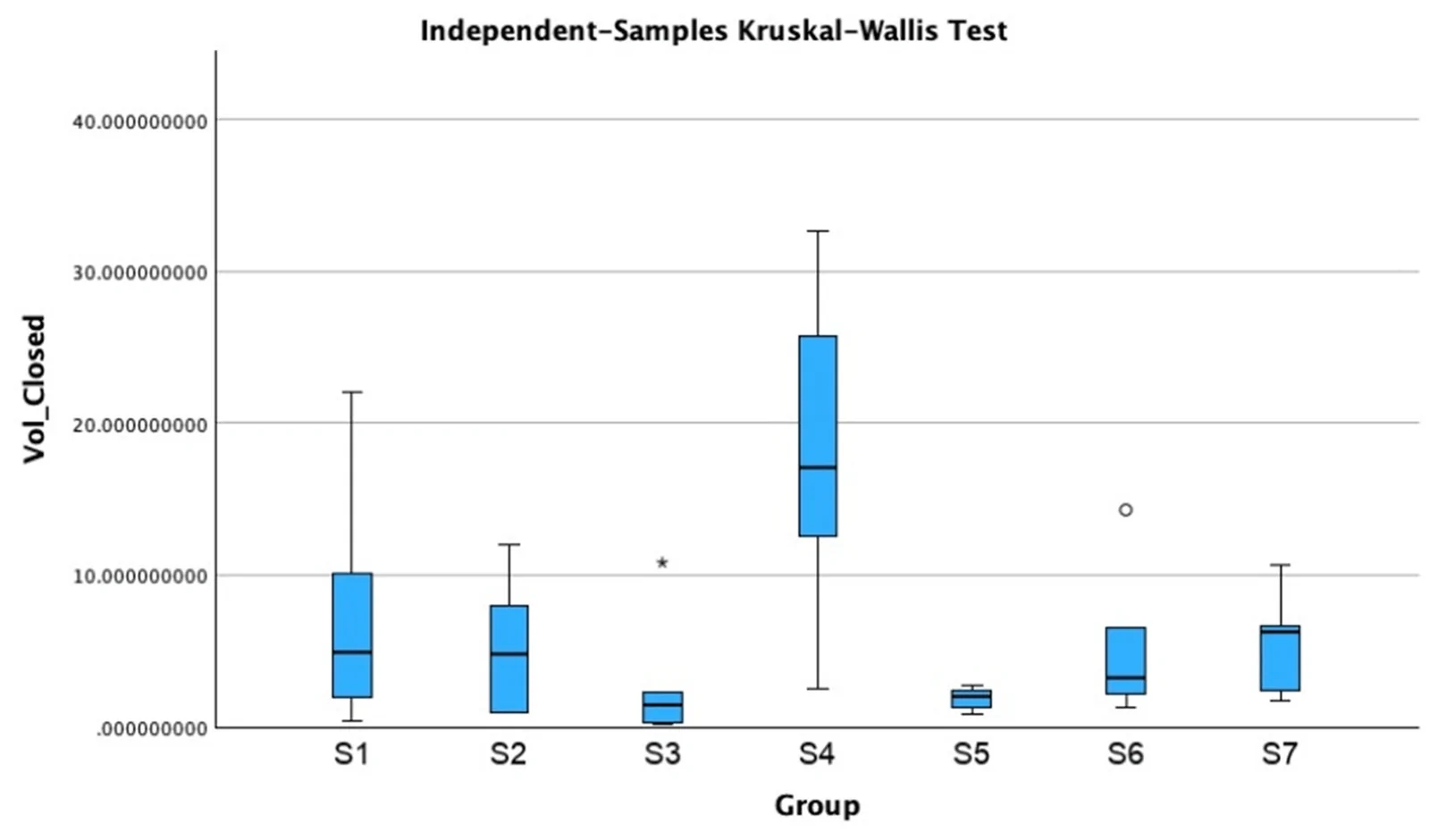
Closed-pore volume (Vol_Closed) across groups S1-S7 in the revised order The Kruskal-Wallis test indicated between-group differences (H(6) = 15.135, p = 0.019), with S4 showing the highest values. Boxes show the middle 50% of values, and the horizontal line marks the median. A circle and an asterisk indicate outliers and extreme outliers, respectively, rather than statistical significance.

**Figure 6:**
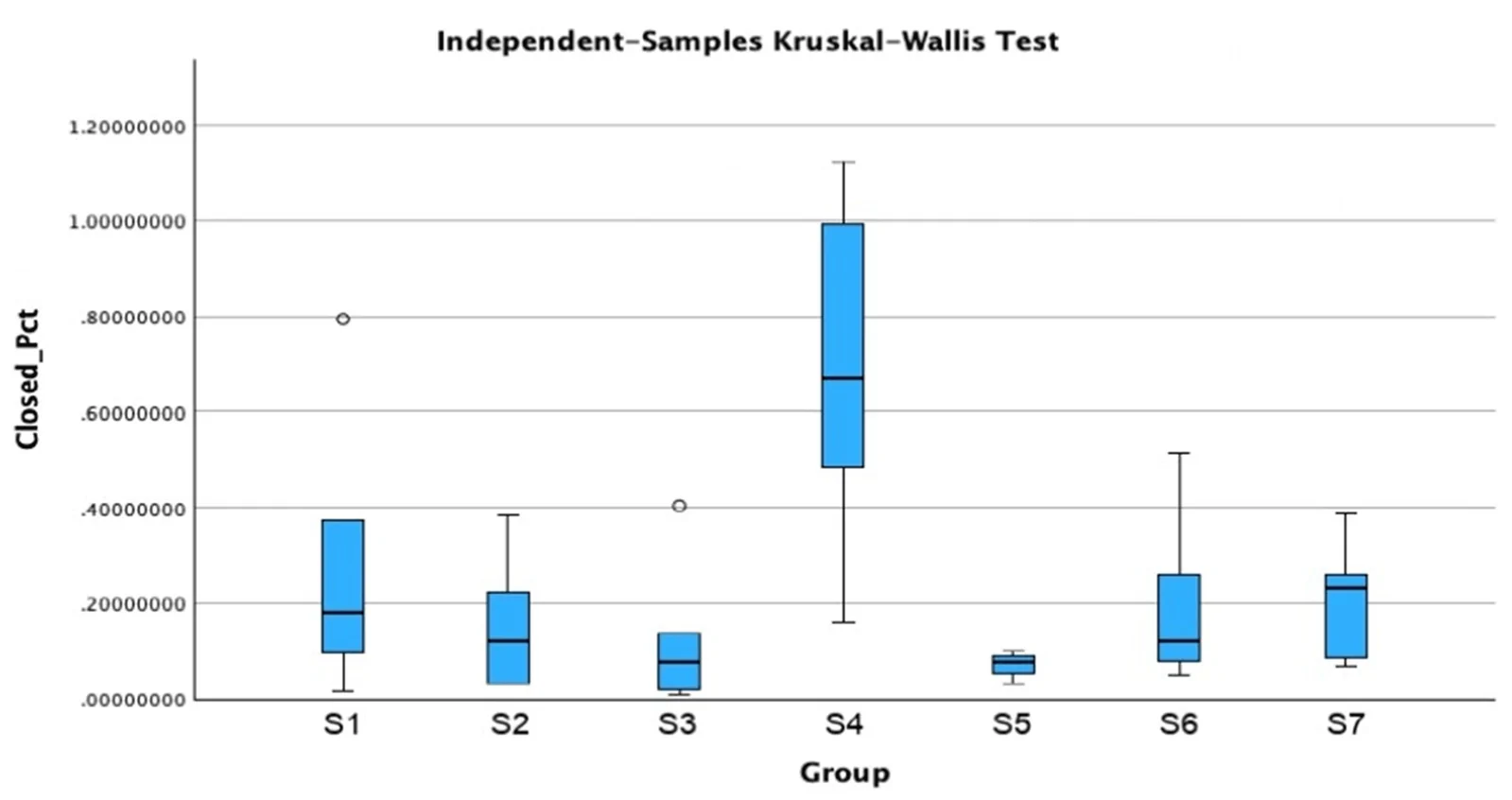
Percentage of closed pores (Closed_Pct) in groups S1-S7 in the revised order The Kruskal-Wallis test indicated between-group differences (H(6) = 15.747, p = 0.015), with S4 showing the highest proportion. Boxes show the middle 50% of values, and the horizontal line marks the median. Circles indicate outliers rather than statistical significance.

For the volume of open pores (Vol_Open) and total pore volume (Total_Vol), the overall Kruskal-Wallis tests indicated between-group heterogeneity (p ≈ 0.025 and p ≈ 0.036, respectively). However, most pairwise comparisons were no longer significant after correction for multiple comparisons; therefore, no specific group was considered consistently superior. Open-porosity percentage (Open_Pct) and total-porosity percentage (Total_Pct) did not differ significantly between groups (p > 0.05; Figure 7).

**Figure 7:**
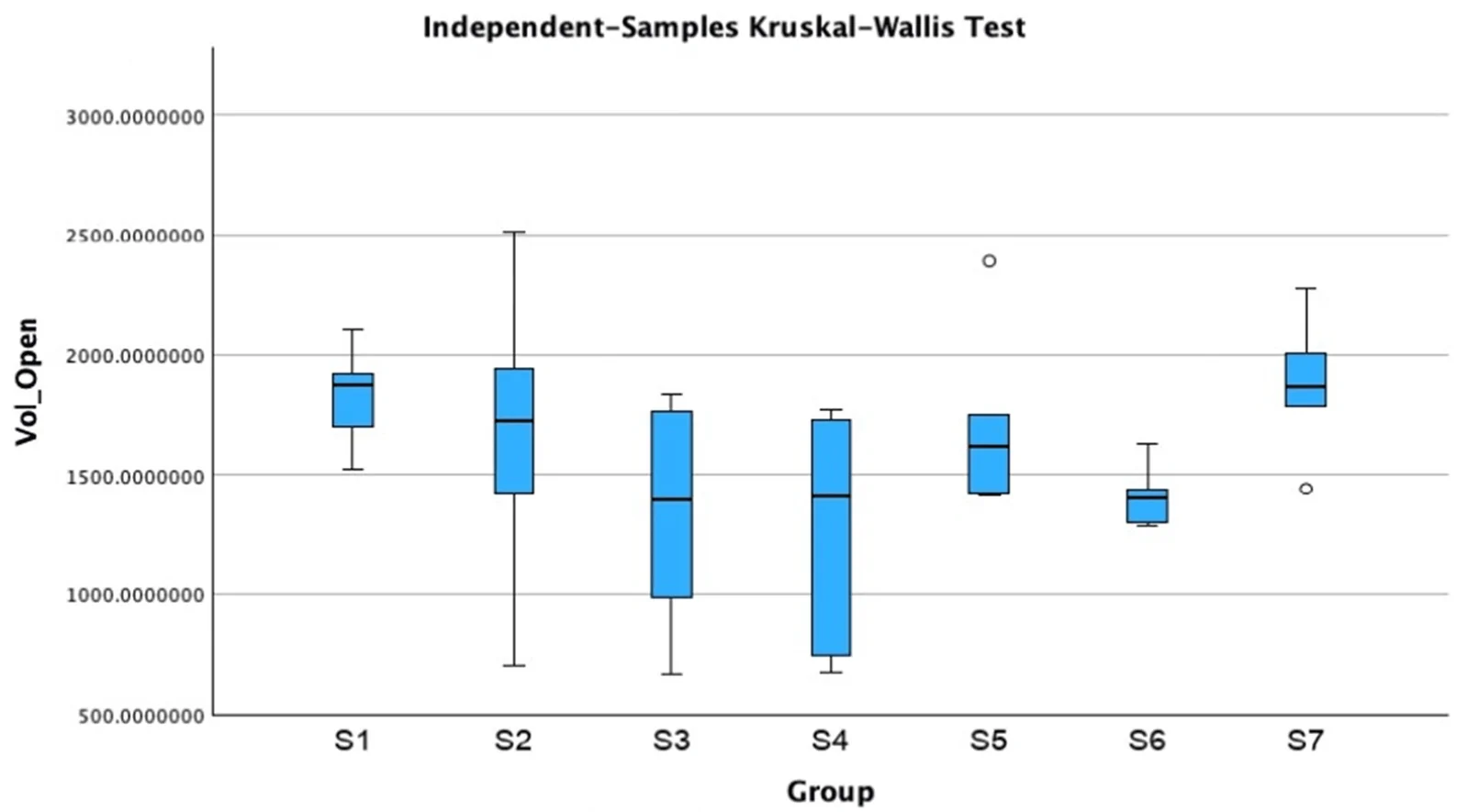
Open-pore volume (Vol_Open) across groups S1-S7 in the revised order The overall Kruskal-Wallis analysis indicated between-group heterogeneity (H(6) = 14.461, p = 0.025), while adjusted pairwise comparisons were mostly non-significant. Boxes show the middle 50% of values, and the horizontal line marks the median. Circles indicate outliers rather than statistical significance.

Descriptively, the antibiotic-free control S1 had the highest mean open-pore volume and total pore volume and one of the highest mean open- and total-porosity percentages. It also had the lowest mean number and surface area of closed pores. Closed-pore volume and closed-pore percentage did not follow a uniform ranking across the antibiotic-containing formulations, so these comparisons should not be interpreted as statistically significant unless supported by the reported multiple-comparison tests.

## Discussion

Raman analysis revealed differences mainly in relative peak intensity and profile at common spectral positions. The bands were compatible with PMMA and previous Raman studies of bone cement [13-17,25]. Although a relationship between Raman intensity and porosity has been reported in porous silicon [26], this finding cannot be directly extrapolated to PMMA bone cement. In the present study, Raman spectroscopy was used only for complementary chemical characterization, and all quantitative porosity conclusions were based on μ-CT.

Several porosity parameters showed statistically significant differences across all seven sample groups. Cement without antibiotics (S1), compared to other specimens with antibiotics either manually or commercially loaded, shows (1) a higher number of pores, total porosity, total pore volume, open porosity, and open-pore volume; (2) a lower number of closed pores, closed porosity, and closed-pore surface area; and (3) variable closed-pore volume. These comparisons describe numerical trends in group means and should not be interpreted as statistically significant differences.

Furthermore, the values for the number of closed pores (N_Closed), the volume of closed pores (Vol_Closed), the surface area of closed pores (Surf_Closed), the percentage of closed pores (Closed_Pct), the volume of open pores (Vol_Open), and the total pore volume (Total_Vol) differed significantly among groups (p < 0.05). This indicates that the microstructure and pore characteristics vary significantly between samples, suggesting differences in pore formation processes or material properties between groups, and possibly even in the mold filling process. Nonparametric Kruskal-Wallis tests confirmed the ANOVA findings, supporting the existence of real differences in data distributions between groups.

In contrast, we did not find statistically significant differences for the percentage of open pores (Open_Pct) and the total percentage of pores (Total_Pct) (p > 0.05). This suggests that although the total and individual pore volumes vary between groups, the relative percentages of open and total pores remain fairly constant. Therefore, groups may differ in terms of the absolute amount of pores, but their ratio to the total material volume does not change significantly. These findings can be used to better understand the pore morphology and potentially help in the selection or optimization of materials according to the application requirements (e.g., permeability, mechanical strength, or even biological behavior).

As shown in Table 3, across the three descriptive manual-versus-commercial pairs (S2-S3, S4-S5, and S6-S7), manually loaded cement had lower mean total- and open-porosity percentages. The mean closed-pore count was lower in the manually loaded gentamicin (S2) and gentamicin-plus-clindamycin (S6) groups but higher in the manually loaded gentamicin-plus-vancomycin group (S4). Total pore volume followed a broadly similar pattern except for the gentamicin comparison (S2 versus S3). Mean closed-pore percentage was higher after manual loading in the gentamicin (S2-S3) and gentamicin-plus-vancomycin (S4-S5) pairs, but not in the gentamicin-plus-clindamycin pair (S6-S7). These trends are descriptive and cannot be attributed solely to loading method because the commercial dual-antibiotic groups used COPAL® (Heraeus Medical GmbH), whereas the corresponding manually loaded groups used PALACOS® R (Heraeus Medical GmbH). These comparisons reflect clinically relevant formulation choices. However, any observed differences may arise from base cement composition, antibiotic content, loading method, or their combined effects. Differences between PALACOS® R and COPAL® therefore cannot be attributed solely to the antibiotic-loading method.

**Table 3:** Comparison of porosity characteristics between loaded and commercially loaded antibiotic groups

Antibiotic	Comparison
Gentamicin S2 vs S3	The commercially loaded group (S3) exhibits higher mean open and total porosity than the manually loaded group (S2). However, the manually loaded group shows a higher mean total pore volume.
Gentamicin and Vancomycin S4 vs S5	The manually loaded group (S4) demonstrates higher mean closed porosity, whereas the commercially loaded group (S5) is characterized by higher mean open and total porosity.
Gentamicin and Clindamycin S6 vs S7	The commercially loaded group (S7) exhibits higher mean open and total porosity, together with a higher mean total pore volume, than the manually loaded group (S6).

Despite the absence of significant differences in total and open porosity among the tested groups, the results revealed statistically significant variations in closed-pore morphology, indicating that internal pore architecture plays a crucial role in material structural organization. Specifically, S7 exhibited the highest number of closed pores, while S4 exhibited significantly increased closed pore volume and surface area, as well as higher closed porosity. Although the presence of an antibiotic substance does not seem to uniformly affect the total porosity, evidence suggests a potential correlation with changes in the closed-pore microstructure. This may ultimately result in differences in hardening, solidification, or microphase distribution during material processing, according to previous studies [27,28]. These findings indicate that materials with similar total porosity may exhibit significant microstructural differences, with potential implications regarding mechanical performance, permeability, and controlled release of incorporated antibiotics. Some pore measurements showed notable variation within groups, particularly the number of closed pores in S4 and S7. This variation may reflect differences in material structure, specimen preparation, and image processing, alongside cement formulation. Although the contribution of each factor could not be assessed separately, these factors provide useful context for interpreting the results. The observed variability and sample size of six specimens per group should therefore be considered when comparing groups. Therefore, qualitative analysis of pore distribution and morphology is an essential complement to conventional porosity evaluation, and future studies should systematically investigate the relationship between microstructural features, mechanical properties, and antibiotic release profiles.

The exploratory XRD analysis showed differences in relative crystalline contribution, but the XRD ranking did not follow the μ-CT porosity ranking. S1 and S7 both showed high mean total porosity, although they presented the highest and lowest XRD indices, respectively. Therefore, no direct relationship between crystalline contribution and pore formation can be concluded. PMMA is predominantly amorphous, and sharp reflections in PMMA bone cement may originate from crystalline radiopacifier or antibiotic phases [18,19]. XRD should consequently be considered complementary phase and structural information rather than a direct measurement of porosity.

This study has several limitations. Firstly, mechanical testing and antibiotic-elution measurements were not performed; therefore, the mechanical and pharmacological consequences of the observed pore structures cannot be determined. Secondly, the sample size was limited, and several pore variables showed large within-group variability. Thirdly, absolute μ-CT values depend on voxel size, region-of-interest selection, segmentation threshold, and minimum pore-size cutoff, which must be fully reported. Fourthly, Raman spectra were acquired from selected surface areas and provide local chemical information only. Fifthly, XRD was based on one pattern per group, and the calculated index was descriptive. Finally, the μ-CT measurements detect pores that can be resolved at a voxel size of approximately 51.63 μm. Smaller pores and narrow connections between pores may not be visible. This may affect the measured porosity and whether pores are classified as open or closed. Therefore, μ-CT may not show all the pore pathways involved in antibiotic transport.

## Conclusions

The tested PMMA formulations differed mainly in closed-pore architecture, including closed-pore number, volume, surface area, and percentage, while no consistent differences were observed in open- or total-porosity percentages. S7 showed the highest closed-pore number, whereas S4 showed increased closed-pore volume and percentage compared with selected groups. Raman spectroscopy provided complementary chemical information and was not used to quantify porosity or crystallinity. Exploratory XRD showed formulation-dependent differences in the crystalline contribution of the complete cement composite, but these differences did not show a consistent relationship with μ-CT porosity. Without evaluating mechanical performance and antibiotic elution, the clinical significance of the observed microstructural differences remains uncertain.
